# Employment Status and Associations with Workability, Quality of Life and Mental Health after Kidney Transplantation in Austria

**DOI:** 10.3390/ijerph17041254

**Published:** 2020-02-15

**Authors:** Galateja Jordakieva, Igor Grabovac, Margarete Steiner, Wolfgang Winnicki, Sabine Zitta, Sinisa Stefanac, Moritz Brooks, Gere Sunder-Plaßmann, Alexander R. Rosenkranz, Jasminka Godnic-Cvar

**Affiliations:** 1 Department of Physical Medicine, Rehabilitation and Occupational Medicine, Medical University of Vienna, Waehringer Guertel 18–20, 1090 Vienna, Austria; galateja.jordakieva@meduniwien.ac.at (G.J.); margarete.steiner@meduniwien.ac.at (M.S.); jasminka.godnic-cvar@meduniwien.ac.at (J.G.-C.); 2 Department of Social and Preventive Medicine, Center for Public Health, Medical University of Vienna, Kinderspitalgasse 15, 1090 Vienna, Austria; sinisa.stefanac@meduniwien.ac.at (S.S.); moritz.brooks@gmx.de (M.B.); 3 Division of Nephrology and Dialysis, Department of Medicine III, Medical University of Vienna, Waehringer Guertel 18–20, 1090 Vienna, Austria; wolfgang.winnicki@meduniwien.ac.at (W.W.); gere.sunder-plassmann@meduniwien.ac.at (G.S.-P.); 4 Division of Nephrology, Department of Internal Medicine, Medical University of Graz, Auenbruggerplatz 27, 8036 Graz, Austria; sabine.zitta@medunigraz.at (S.Z.); alexander.rosenkranz@medunigraz.at (A.R.R.); 5 Institute of Outcome Research, Center for Medical Statistics, Informatics and Intelligent Systems, Medical University of Vienna, Spitalgasse 23, 1090 Vienna, Austria

**Keywords:** quality of life, WHOQOL, kidney transplant, workability, WAI, return to work

## Abstract

Kidney transplantation (KTx) in end-stage renal disease is associated with a significant increase in quality of life (QoL) and self-perceived health, optimally leading to the maintenance of employment or return to work (RTW) in working-age patients. The aim of this study was to assess individual factors including the QoL and mental health of kidney transplant recipients (KTRs) associated with employment after transplantation. A cross-sectional study including working-age patients with a history of KTx after 2012 was conducted at two Austrian study centers (Vienna and Graz). Brief Symptom Inventory (BSI-18), World Health Organization Quality of Life (WHOQOL-Bref) and Workability Index (WAI) were assessed along with detailed questionnaires on employment status. Out of *n* = 139 KTRs (43.2 ± 9.07 years; 57.6% male), 72 (51.8%) were employed. Employed patients were more frequently in a partnership (*p* = 0.018) and had higher education levels (*p* = 0.01) and QoL scores (<0.001). Unemployed KTRs reported fatigue and mental health issues more often (*p* < 0.001), and had significantly higher anxiety, depression and somatization scores (BSI-18). In unadjusted logistical regression, workability score (WAS; odds ratio (OR) = 3.39; 95% confidence interval (CI) = 1.97–5.82; *p* < 0.001), partnership (OR = 5.47; 95% CI 1.43–20.91; *p* = 0.013) and no psychological counseling after KTx (OR = 0.06; 95% CI = 0.003–0.969; *p* = 0.048) were independently associated with employment. Self-assessed mental health, workability and QoL were significantly associated with employment status after KTx. Thus, in order to facilitate RTW after KTx in Austria, vocational rehabilitation and RTW programs addressing KTRs should focus on increasing social support and care for their mental health.

## 1. Introduction

Each year, approximately 600–800 patients with end-stage renal disease (ESRD) throughout Austria are listed for kidney transplantation (KTx). Country-wide, organ transplantations are carried out at specialized centers located in Vienna, Graz, Linz, and Innsbruck [1], with a total of over 400 KTx being performed each year. In kidney transplant recipients (KTRs), vocational rehabilitation is particularly desirable, as reports over the past decade indicate that the overwhelming majority (60–70%) of transplant recipients in Austria are of working age [2]. Returning to normality and “full-life” participation, e.g., working and community life, are an essential need of most KTRs [3]. As employment is known to have several salutogenetic effects, return to work (RTW) is associated with higher quality of life (QoL) as well as better emotional and mental health in these patients [4,5,6,7]. Furthermore, employment post-KTx was associated with overall KTR and allograft survival [8]. The improved outcomes observed amongst employed KTRs were attributed to a higher adherence to treatment, better health maintenance practices and higher levels of physical activity, along with a greater desire to participate in an active life [8]. A crucial psychological factor in this context appears to be self-efficacy [9], i.e., the KTR’s belief in their own capacity to successfully deal with a challenging situation, besides other aspects such as effective post-transplant adaptation [10] and nursing social contacts [11]. Higher self-efficacy can positively influence mental health and QoL [12] in KTRs and was identified as a protective factor in regard to stress, anxiety, depression and somatization [13]. KTRs with high self-efficacy showed better adherence to therapy [14] and improved functional coping behavior, leading to more favorable outcomes post-KTx [13]. It is important to note that mental health is not only directly linked to QoL [6] but may also impact post-transplant morbidities associated with unemployment post-KTx, e.g., fatigue [15,16]. Facilitation of RTW was amongst the main predictive factors for improved QoL in KTRs [17], along with the interrelated reduction in adverse KTx-associated health events. In light of this knowledge, the assessment of individual factors geared towards the facilitation of employment is important in KTRs [18,19]. 

Excellent rehabilitation results have been achieved after KTx, particularly when compared to other solid organ transplantations [18]. Patients with a long-term functioning allograft (≥10 years) were shown to reach “pre-illness levels of activity” in terms of workability [20]. A large cross-sectional Finnish study found a general employment rate of about 40% in patients with a functioning transplant, with approximately 30% taking up full-time employment following KTx [21]. Still, studies show a high degree of fluctuation in employment status of KTR. In a recent systematic literature review, sociodemographic aspects, physical and psychological comorbidities, as well as factors related to the transplant process, were identified as important determinants of successful employment and RTW in KTRs [22,23,24,25]. More specifically, sociodemographic predictors of RTW included pre-transplant employment [26], type of work [27], shorter unemployment duration after KTx, being married, younger age and higher educational level [7,16,18]. Further, a shorter dialysis period preceding transplantation, minimal invasive kidney surgery (e.g., laparoscopic donor nephrectomy) and living kidney donor were also associated with favorable RTW outcomes after transplantation [24]. In contrast, diabetes and depression were particularly unfavorable comorbidities in regard to RTW rates at 12 months after KTx [26].

In Austrian social legislation, based on the subsequent need for lifelong immunosuppression, even optimal KTx outcomes result in an “earning capacity reduction” status. This disability status, targeted at workers with chronic diseases, is associated with some extent of legal protection, resulting in social and vocational benefits. Depending on further health limitations, e.g., concomitant physical and psychological comorbidities, even higher degrees of disability adjudication are possible after KTx. While this legislation provides relevant support regarding (re)employment, understanding individual and health-related needs of KTRs is an essential foundation for commencing a successful RTW process.

Based on these considerations and the belief that RTW is one of the essential factors in regaining full life quality and social engagement, this study was designed to provide an update on employment status, QoL, self-reported mental health, workability and associated factors in patients after kidney transplantation.

## 2. Materials and Methods

### 2.1. Participants

Our study participants were patients visiting the outpatient nephrology clinic in two study centers between 2016 and 2019. Patient recruitment was done at two centers: The Medical University of Vienna (Medizinische Universität Wien) and the University Hospital Graz (Universitätsklinikum Graz). All participants between 18 and 55 years of age, having had their KTx performed after 2012 at least 6 months prior to inclusion, and with sufficient knowledge of the German language, were included in the study only after giving their informed consent. In order to minimize potential selection bias, all participants who matched the inclusion criteria were consecutively approached when they came to their regularly scheduled nephrological check-ups.

Inclusion Criteria

- Male and female post-KTx outpatients
- KTx performed after 2012 and at least 6 months prior to inclusion
- Age between 18 and 55 years
- Sufficient knowledge of German language
- Signed declaration of informed consent

Exclusion Criteria

- Dyspnea NYHA classification III-IV
- Unsigned declaration of consent
- Major physical and psychological impairments
- Inadequate compliance for answering the questionnaires
- Significant barrier of language

### 2.2. Methods

The study was designed as a multicenter cross-sectional study. Patients’ data were gathered via paper-based questionnaires filled in before or after their nephrology consultations. The study aims were explained to the patients and those who agreed to participate were taken to an allocated area, where they could fill out the survey in privacy. It took each participant approximately 15 min to fill in the questionnaires. A member of the study team remained within each patient’s reach at all times in order to clear up any potential issues. The filled out questionnaires were placed in sealed envelopes and stored in a locked cabinet and later delivered to the analysis team who established the database and analyzed the data. The patients’ identity was not known to the analysis team and could thus not link individual questionnaires to the individual patients.

### 2.3. Questionnaires

For the purposes of the study, a questionnaire set composed of 116 items divided into 5 parts was created, compiled of standardized and non-standardized instruments. The questionnaire set consisted of single- and multiple-choice questions, as well as open-end questions.

1. Sociodemographic and general information about the health status was obtained in the first part of the questionnaire. This included questions on various social and demographic aspects (employment status, education level, family members) and questions on health (height and weight, smoking status, alcohol consumption, physical activity levels).
2. Occupational history included questions on the working life, including all working experiences, types of occupational activity performed, as well as subjective satisfaction with the working conditions.
3. Brief Symptoms Inventory 18 (BSI-18) is a self-administered questionnaire comprising of a list with 18 symptoms where the participants determined the intensity of symptoms during the past 7 days on a Likert type scale (0–4; 0 = not at all, 4 = very much). Scores are calculated by sum scores. The BSI-18 has 3 subscales: depression, anxiety and somatization and the Scale Global Severity Index. This questionnaire was chosen as previous multiple application studies indicated that BSI-18 is a suitable instrument for measuring psychological distress. A validated German language version of the questionnaire was used [28].
4. Workability Index (WAI) is a widely used instrument in occupational health to assess the workability and covers several dimensions of workability including current workability in relation to job demands and their lifetime best level of workability, number of comorbidities and the estimated impairment arising from disease or other limiting conditions, amount of sick leave and prognosis of one’s workability. The items are a variation of single-, multiple-choice and Likert-type scale questions, with a score being derived by summation. Given that the WAI can only be applied in subjects who are currently employed, we also calculated the Workability Score (WAS) which is a subscore derived from a single item of the WAI, which is valid and suitable for both currently employed as well as unemployed participants [29].
5. World Health Organization Quality of Life Questionnaire Bref is a shorter form of the WHOQOL-100. The items are divided into 4 domains (physical, psychological, social relationships and environment) that are scored on a Likert-type scale (ranging from 1 to 5, 1 indicating low or negative perceptions and 5 high or positive ones). The scores are a sum, with some items being not scaled in a positive direction. We used the official German translation of the World Health Organization Quality of Life (WHOQOL-Bref) [30].

### 2.4. Statistical Analysis

Descriptive statistics were performed for each variable. Quantitative variables are presented as mean values and standard deviation, while qualitative variables are presented as frequencies and percentages. Differences in frequencies of categorical variables were calculated using the Chi-square test with Fischer exact, while differences between mean values were tested by the t-test for unpaired samples.

A multivariate logistic regression model was performed to determine which variables were associated with employment status. Based on univariate analysis, all variables with a cut off value of *p* < 0.2 were included in the multivariate model. All *p*-values below 0.05 were considered statistically significant. The analyses were performed using the SPSS 24.0 statistical software (IBM Corp., Armonk, NY, USA).

### 2.5. Ethical Consideration

The study was approved by the Ethical Committee of the Medical University of Vienna. The study was conducted in accordance with the Helsinki Declaration and the principles of Good Clinical Practice (EK Number 1233/14). Informed consent was obtained from all participants before inclusion in the study.

## 3. Results

A total of 139 patients (43.2 ± 9.07 years; 57.6% male) agreed to participate in the study. The study sample was stratified by employment status, with 51.8% of participants reporting being employed. Statistically significant differences were observed regarding education level, i.e., a higher education level was found among employed subjects compared to unemployed subjects, and partnership status, i.e., employed subjects reported living in partnership more often than unemployed. The sociodemographic characteristics of the participants are presented in Table 1.

Regarding health-associated variables, significant differences in employment status were only found in terms of experiencing fatigue and participating in psychological counseling after KTx (see Table 2).

In employed subjects, the mean Workability Index score was 36.82 (standard deviation (SD) = 6.13), with 61% of scores being in the “good” range of the workability category. Significant differences were found in the workability score, whereby employed participants had the mean value of WAS twice as high as the unemployed participants. Similarly, differences were found in all subscales of the BSI-18, as unemployed participants scored higher (indicating more psychological distress) on somatization, depression, anxiety and Global Severity Index compared to employed participants (Table 3).

Employed participants scored higher in the WHOQOL-Bref questionnaire as well—significant differences were found in the overall score, the global quality of life and the physical domain. Although in employed subjects higher scores were found in all domains of the WHOQOL-Bref, they failed to reach statistical significance (Table 4).

Psychological counseling received after KTx was associated with 95% higher odds of being unemployed. Each incremental increase in the WAS was associated with more than three times higher odds of being employed.

## 4. Discussion

In our cross-sectional study sample of outpatient KTRs, 51.8% were currently employed and 22.8% were early-retired, which is in line with recent reports from other European countries [21,24,31]. While patients with a successful KTx re-transplant have higher working capacity than those who are forced to return to dialysis due to transplant rejection [32], our findings for the Viennese subgroup show a decrease in employment among recipients of consecutive KTx. Almost 52% of patients reported working after the first and 38.5% after the second kidney transplant, but no participant was working after multiple kidney transplants (>3 KTx). Similar percentages of patients working after KTx (56.2%) were found 12 months post-transplantation in the Swiss Transplant Cohort [24]. A Dutch study, published in 2011, reported employment in 67% of patients post-KTx, with 30% of working KTRs still receiving disability benefits [31]. A large study on KTRs in the USA found that rates of employment seem stable within a 5-year follow up period (47% at year 1 and 43% at year 5) in patients with private insurance, while the rates were relatively lower in those without private insurance, reported between 12% and 16% [33]. An even lower rate of employment (8%) was found in patients who were not working at the time of KTx [33]. Another US study found that only 35.5% were employed 12 months post KTx, with 74.5% receiving disability benefits [34]. A retrospective cohort study in Brazil showed even lower RTW rates of only 26%, which the authors attributed to national disability pension policies [35]. Several of these and other studies [7,22] identified a higher educational background as a facilitator of RTW.

Higher educational levels were significantly associated with employment in our patient sample. Most of our patients were working in the private sector (60.6%), possibly indicating socioeconomic and/or professional incentives (e.g., income, responsibility) resulting in higher motivation for RTW. The majority of our participants rated their workability as good or very good (61%), which is in line with reports by other authors [31]. As expected, significantly better ratings throughout all WAI categories were found amongst employed subjects at the time of inclusion, while only 9% of unemployed KTRs rated their workability to be above moderate. The single-item WAS, comparing self-estimated current workability to best lifetime workability, was most significantly associated with current employment, with a three times odds increase for each gain in total score. While it might not replace the multi-item WAI [36], our findings indicate that WAS might serve as a sufficient screening tool in KTRs.

In our study, unemployed subjects scored significantly higher on the somatization, depression and anxiety subscales of the Brief Symptoms Inventory 18. Experiencing fatigue was reported by 57.5% of our study participants and was more common amongst unemployed KTR. Psychological health aspects and positive self-perceived workability are essential for employment after KTx [18]. The resumption of employment in transplant patients can be hindered by perceived physical appearance, impairment of sexuality, stress and anxiety [37]. Additionally, KTx may lead to specific mental burdens, such as feelings of guilt towards the donor, dependency on the living donor and graft loss fears [38]. A recent systematic review emphasized the negative impact of depressive symptoms on RTW rates after KTx [26], potentially based on poorer neuropsychological performance and fewer predicted working hours in employed patients [39]. Our unemployed participants were more commonly taking psychological counseling after KTx than the employed. While this might be interpreted as being a limiting factor for RTW, it appears more likely that severe mental health issues drive these patients to seek professional assistance. Due to our cross-sectional study design and despite asking about psychological counseling before KTx, it remains unclear whether these particular patients had suffered mental health issues prior to KTx as well, thereby negatively impacting their post-transplant employment [22]. Despite higher QoL and health-related QoL rates in KTRs compared to ESRD [5,40,41,42,43,44], psychological burden prior to transplantation still appears to be a significant QoL predictor thereafter [43]. While we found no significant differences between employed and unemployed participants regarding the psychological QoL domain, unemployed subjects had significantly lower overall QoL scores as measured by the WHOQOL-Bref. Thus, the early assessment of emotional stress factors, both before and after kidney transplantation, is important in order to provide adequate psychological support during rehabilitation and RTW [38,45]. 

In line with other authors [22,46,47], we also found that partnership status, particularly living in a relationship or being married, was more commonly reported among employed KTR. Taken together, patients in partnerships were five times more likely to be employed compared to singles or divorcees. Despite inconsistency in previous reports [22], gender had no impact on employment after KTx in our study. In contrast to partnership status in general, caring for underage children or family members was not significantly associated with employment, implying that the importance of social support outweighs even in settings with multiple responsibilities.

The strong association of partnership status with employment stresses the importance of social support in the context of RTW. While private relationship dynamics are largely beyond the sphere of professional influence, early psychosocial counseling and health-related support could contribute to individual support and encourage patients’ self-motivation to return to work, as suggested by other studies [48,49]. An explicit need for guidance related to RTW, as well as health-related and emotional coping is reported in KTR, who consider the state of post-transplant counseling as suboptimal [50]. In order to maintain the employability of the KTR, a professional assessment of individual measures is needed, along with an efficient personnel reintegration policy. Multidisciplinary approaches to patient education and psychosocial support are facilitators of community reintegration in KTRs [22,49].

## 5. Limitations

While we found clear associations between sociodemographic, QoL and mental health factors with employment after KTx, the direction of causality cannot be determined due to the cross-sectional design of our study. Furthermore, the lack of prospective analysis limited our ability to assess any future RTW and/or loss of employment in our patient group. Longitudinal studies encompassing pre-transplant employment and workability data could also significantly contribute to the identification and harnessing of relevant predictors in regard to post-transplant outcomes. Lastly, due to the recruitment of subjects at an outpatient clinic for KTR, some extent of selection bias, i.e., the exclusion of patients with worse health status and low adherence, has to be considered, particularly regarding RTW rates. Thus, our results may not be generalized to the entire KTx population in Austria.

## 6. Conclusions

In our study, living with a partner, having a higher education status and better quality of life was more common amongst employed KTRs. These findings are in line with the scientific literature, indicating the value of social support and favorable impact of employment on QoL after KTx. The goal of organ transplantation is not limited to the extension of life span but aims at the improvement of well-being, QoL and participation. Comprehensive care after transplantation should encompass the enablement and facilitation of RTW. Supportive measures enabling the reintegration of kidney transplant recipients into the workplace are associated with both positive individual outcomes and socio-economic effects in an aging society, as is the case in Austria and other European countries. Vocational rehabilitation and integration programs should encompass the evaluation of emotional and psychological needs in regard to RTW and working environment. As mental health-related issues, such as somatization, depression and anxiety, but also fatigue, were more common in unemployed subjects, psychosocial services supporting mental health and disease processing should be considered even before KTx in order to counteract psychological consequences, such as avoidance of participation, at an early stage. Our study is the first to assess employment post-KTx and its interrelations with subjective workability, QoL and mental health in Austria. Country-specific aspects regarding RTW in KTRs can provide additional insights into potentially modifiable limitations to employment after KTx in these patients.

## Figures and Tables

**Table 1:** Sociodemographic data. Analysis of sociodemographic data in all participants and stratified by current employment status.

Variables		Total *n =* 139 (*n*, %)	Employed (*n* = 72)	Unemployed (*n* = 67)	*p*-Value
Study Center					0.319
	Graz	33 (24.6)	20 (27.8)	13 (19.4)	
	Vienna	106 (76.3)	52 (72.2)	54 (80.6)	1
Gender					
	Male	80 (57.6)	41 (56.9)	39 (58.2)	
	Female	59 (42.4)	31 (43.1)	28 (41.8)	
Education Level					**0.01**
	Primary	45 (32.4)	15 (20.8)	21 (31.3)	
	Vocational training	48 (34.5)	27 (37.5)	30 (44.8)	
	Secondary	31 (22.3)	22 (30.6)	9 (13.4)	
	Tertiary	15 (10.8)	8 (11.1)	7 (10.4)	
Partnership status					**0.018**
	Single	37 (26.6)	17 (23.6)	20 (29.9)	
	Partnership	22 (15.8)	15 (20.8)	7 (10.4)	
	Married	56 (40.3)	33 (45.8)	23 (34.3)	
	Divorced	23 (16.5)	6 (8.3)	17 (25.4)	
	Widowed	1 (0.7)	1 (1.4)	0	
Underage children in household					0.273
	Yes	44 (31.7)	26 (36.1)	18 (26.9)	
	No	94 (67.6)	45 (62.5)	49 (73.1)	
	Missing		1 (1.4)	0	
Providing care for family members					0.599
	Yes	16 (11.5)	7 (9.7)	9 (13.4)	
	No	122 (87.8)	64 (88.9)	58 (86.6)	
	Missing	1 (0.7)	1 (1.4)	0	
Employed					
	Yes	72 (51.8)			
	No	67 (48.2)			
Employment					
	Full time	69.4 (50)			
	Part time	26.4 (19)			
	Marginally	4.2 (3)			
Place of employment					
	Private company	43 (60.6)			
	Family owned	8 (11.3)			
	Public sector	20 (28.2)			
Early retired					
	Yes	31 (22.3)			
	No	104 (74.8)			
	Missing	4 (2.9)			
Type of occupational activity at last job					0.059
	Mostly physical work	12 (20.3)	5 (50.0)	7 (14.3)	
	Mostly intellectual work	15 (25.4)	1 (10.0)	14 (28.6)	
	Both in equal measure	32 (54.2)	4 (40.0)	28 (57.1)	
Years employed (Mean, SD)		20.40 (10.67)	21.49 (9.74)	19.23 (11.54)	0.229
Age in years (Mean, SD)		43.20 (9.07)	42.49 (8.47)	43.95 (9.67)	0.346

Chi-square test with Fischer exact was done for categorical variables and independent samples t-test for metric variables; SD—standard deviation. *p* < 0.05 values are presented as bold text.

**Table 2:** Health-related outcomes. Analysis of health data in all participants and stratified by current employment status.

Variables		Total *n =* 139 (*n*, %)	Employed (*n* = 72)	Unemployed (*n* = 67)	*p*-Value
Height in cm (Mean, SD)		171.59 (9.86)	172.32 (9.28)	170.81 (10.45)	0.386
Weight in kg (Mean, SD)		76.70 (16.33)	77.24 (16.83)	76.13 (15.88)	0.693
BMI (kg/m^2^) (Mean, SD)		25.98 (4.79)	25.88 (4.57)	26.09 (5.05)	0.799
Smoking status					0.427
	Smoker	33 (23.7)	15 (20.8)	18 (26.9)	
	Non-smoker	105 (75.5)	57 (79.2)	48 (71.6)	
	Missing	1 (0.7)	0	1 (1.5)	
Drinking alcohol					0.054
	Yes	52 (37.4)	33 (45.8)	19 (28.4)	
	No	85 (61.2)	39 (54.2)	46 (68.7)	
	Missing	2 (1.4)	0	2 (3.0)	
Alcohol consumption frequency					1.146
	No alcohol	85 (62.0)	39 (54.2)	46 (70.8)	
	Occasionally (few times monthly)	43 (31.4)	26 (36.1)	17 (26.2)	
	Frequently (few times a week)	8 (5.8)	6 (8.3)	2 (3.1)	
	Daily consumption	1 (0.7)	1 (1.4)	0	
Regular exercise					0.469
	Yes	76 (54.7)	37 (51.4)	39 (58.2)	
	No	63(45.3)	35 (48.6)	28 (41.8)	
Exercise frequency					0.813
	Less than 4 times a month	5 (6.9)	3 (8.3)	2 (5.6)	
	Once a week	15 (20.8)	9 (25.0)	6 (16.7)	
	Several times a week	39 (54.2)	18 (50.0)	21 (58.3)	
	Daily	13 (18.1)	6 (16.7)	7 (19.4)	
Surgery number (only Vienna)					0.834
	1. KTx	85 (80.2)	44 (84.6)	41 (75.9)	
	2. KTx	13 (12.3)	5 (9.6)	8 (14.8)	
	3. KTx	4 (3.8)	2 (3.8)	2 (3.7)	
	5. KTx	1 (0.9)	0	1 (1.9)	
	KTx + PTx	3 (2.8)	1 (1.9)	2 (3.7)	
Offered psychological support before surgery					0.734
	Yes	64 (46.0)	32 (44.4)	32 (47.8)	
	No	73 (52.5)	39 (54.2)	34 (50.7)	
	Missing	2 (1.4)	1 (1.4)	1 (1.5)	
Offered psychological support after surgery					0.376
	Yes	50 (36.7)	23 (31.9)	27 (40.3)	
	No	86 (61.2)	47 (65.3)	39 (58.2)	
	Missing	3 (2.1)	2 (2.8)	1 (1.5)	
Taken psychological support before surgery					0.098
	Yes	30 (21.6)	11 (15.3)	19 (28.4)	
	No	108 (77.7)	60 (83.3)	48 (71.6)	
	Missing	1 (0.7)	1 (1.4)	0	
Taken psychological support after surgery					0.019
	Yes	22 (15.8)	6 (8.3)	16 (23.9)	
	No	116 (83.5)	65 (90.3)	51 (76.1)	
	Missing	1 (0.7)	1 (1.4)	0	
Experiencing fatigue					<0.001
	Yes	77 (55.4)	44 (61.1)	53 (79.1)	
	No	57 (41.0)	24 (33.3)	13 (19.4)	
	Missing	5 (3.6)	4 (5.6)	1 (1.5)	

Chi-square test with Fischer exact was done for categorical variables and independent samples t-test for metric variables; BMI—body mass index; KTx—kidney transplant; PTx—pancreas transplant.

**Table 3:** Workability, quality of life, mental health symptoms and employment status. Descriptive analysis of Work Ability Index (only currently employed), Brief Symptoms Inventory for mental health issues and quality of life scores as measured by the World Health Organization’s Quality of Life Questionnaire.

**Workability Score**				
**Variables**	**Total *n =* 139 (*n*, %)**	**Employed (*n* = 72)**	**Unemployed (*n* = 67)**	***p*-Value**
WAS (Mean, SD)	5.83 (2.84)	7.82 (1.55)	3.69 (2.30)	<**0.001**
WAS Category				<**0.001**
Bad	58 (41.7)	4 (6.0)	54 (80.6)	
Moderate	28 (20.1)	21 (31.3)	7 (10.4)	
Good	33 (23.7)	28 (41.8)	5 (7.5)	
Very good	15 (10.8)	14 (20.9)	1 (1.5)	
**Brief Symptoms Inventory**				
**Variables**	**Total *n =* 139 (*n*, %)**	**Employed (*n* = 72)**	**Unemployed (*n* = 67)**	***p*-Value**
Global Symptoms Index (Mean, SD)	11.72 (12.50)	6.33 (7.51)	17.51 (14.15)	<**0.001**
Somatization	4.12 (4.67)	2.10 (2.67)	6.28 (5.35)	<**0.001**
Depression	3.58 (4.53)	1.75 (3.02)	5.54 (5.05)	<**0.001**
Anxiety	4.03 (4.73)	2.49 (3.26)	5.69 (5.46)	<**0.001**
**WHO Quality of Life Questionnaire**				
**Variables**	**Total *n =* 139 (*n*, %)**	**Employed (*n* = 72)**	**Unemployed (*n* = 67)**	***p*-Value**
Quality of life score	76.37 (24.71)	84.68 (21.73)	67.31 (24.72)	<**0.001**
Global QoL	51.04 (29.75)	58.08 (30.43)	43.36 (27.19)	**0.003**
Physical domain	44.56 (21.42)	49.23 (23.28)	39.47 (18.01)	**0.006**
Psychological domain	49.66 (24.43)	52.90 (25.60)	46.14 (22.76)	0.105
Social relationship domain	56.15 (29.65)	58.06 (30.51)	54.05 (28.76)	0.429
Environment domain	58.06 (28.75)	62.55 (30.90)	53.16 (25.53)	0.053

Chi-square test with Fischer exact for categorical variables and an independent samples t-test for metric variables; WAS—workability score; WHO—World Health Organization; QoL—quality of life. *p* < 0.05 values are presented as bold text.

**Table 4:** Variables associated with current employment in kidney transplant recipients. Unadjusted logistical regression model assessing variables which are associated with current employment in kidney transplant recipients (employment status as dependable variable). Variables included in the model were chosen based on the univariate analysis with a cutoff point of *p* < 0.2.

Variables	Odds Ratio	95% Confident Interval		*p*-Value
Education				
Vocational (Ref)	1			
Primary	0.335	0.059	1.9	0.217
Secondary	0.616	0.256	10.009	0.616
Tertiary	8.849	0.424	184.733	0.16
Partnership				
Single (Ref)	1			
Partnered	5.47	1.43	20.911	**0.013**
Psychological help before surgery				
No (Ref)	1			
Yes	1.386	0.207	9.283	0.737
Psychological help after surgery				
No (Ref)	1			
Yes	0.055	0.003	0.969	**0.048**
Alcohol consumption				
No (Ref)	1			
Yes	1.882	0.485	7.304	0.361
WAS	3.369	7.979	5.827	<**0.001**
Global Symptoms Index	0.769	0.272	0.436	0.367
BSI Somatization	1.148	0.63	2.094	0.652
BSI Depression	0.959	0.499	1.844	0.900
BSI Anxiety	1.766	0.924	3.375	0.085
WHOQOL-Bref Score	0.978	0.933	1.025	0.356
Global Quality of Life	0.986	0.922	1.056	0.693
Physical domain	1.073	0.97	1.188	0.172
Psychological domain	0.915	0.835	1.002	0.055
Environmental domain	1.022	0.95	1.099	0.561

BSI—brief symptom inventory; WHOQOL-Bref—World Health Organization Quality of Life. *p* < 0.05 values are presented as bold text.
